# Unique Genetic Characteristics and Clinical Prognosis of Female Patients with Lung Cancer Harboring RET Fusion Gene

**DOI:** 10.1038/s41598-020-66883-0

**Published:** 2020-06-25

**Authors:** Zhixin Qiu, Bingwei Ye, Ke Wang, Ping Zhou, Shuang Zhao, Weimin Li, Panwen Tian

**Affiliations:** 1 Department of Respiratory and Critical Care Medicine, West China Hospital, Sichuan University, Chengdu, 610041 P.R. China; 2 Georgia Cancer Center, Augusta University, Augusta, GA 30912 USA; 3 Department of Pathology, West China Hospital, Sichuan University, Chengdu, P.R. China; 4 Lung Cancer Treatment Center, West China Hospital, Sichuan University, Chengdu, Sichuan Province P.R. China

**Keywords:** Cancer, Medical research, Molecular medicine

## Abstract

Objectives: Since no report on the genetic characteristics of RET fusions in female patients with lung cancer is available, this study revealed the genetic and prognostic characteristics of female patients with lung cancer harboring RET fusion gene for the first time. Materials and Methods: The molecular portfolios of 1,652 patients with lung cancer who underwent targeted next-generation sequencing for screening candidate oncogenic drivers in their histological specimens from January 2016 to December 2018 were investigated in this study. Results: RET fusions were identified in 23 cases, 15 females [2.2% (15/685)] and eight males [0.9% (8/902)]. The most common fusions were KIF5B–RET in females [80% (12/15)] and CCDC6–RET in males [50% (4/8)], along with some rare RET fusions, including SLC39A8–RET, ITIH2–RET, FYCO1–RET and SLC25A36–RET in females, and MIR3924–RET, ZBTB41–RET and ITGA8–RET in males. Interestingly, the highly positive, moderate positive, and negative rates of PD–L1 staining in females were 33.3%, 8.3% and 58.3%, respectively; whereas those in males were 0%, 57.1% and 42.9%. Additionally, the progression-free survival (PFS) of stage IV patients was comparatively shorter in females, shown by the medians of 4.0 months in females and 6.0 months in males (*P* = 0.029). A 43-year-old female patient with metastatic lung adenocarcinoma, who harbored KIF5B–RET fusion and had highly positive PD–L1 staining, received nivolumab as second-line treatment. A partial response was achieved and remained for more than five months. Conclusion: Unique genetic characteristics and poor prognosis are found in female patients with lung cancer harboring RET fusion gene. Immune checkpoint inhibitors are a potential option for patients with high expression of PD–L1.

## Introduction

Rearrangement during transfection (RET), was identified by Takahashi *et al*. in 1985 as a proto-oncogene that underwent rearrangement during the transfection of DNA extracted from human T-cell lymphoma into NIH-3T3 cells^1^. Physiologically playing an important role in the development of neurons and kidneys, RET is recognized as the growth factor receptor of the glial cell line-derived neurotrophic factor (GDNF) family. RET fusions, one of the rare driver genes in lung cancer, have been detected in 1–2% of all lung cancers and in approximately 1.6% of Chinese non-small cell lung cancers (NSCLC)^2,3^. The most common RET fusion partners are kinesin family 5B (KIF5B) and coiled-coil domain containing 6 (CCDC6), which have been reported in 70–90% and 10–25% of cases, respectively^2,4–6^. Fusion genes play a major role in the pathogenesis of lung cancers, and the discovery of microtubule-associated protein-like 4–anaplastic lymphoma kinase (EML4–ALK) fusion kinase in 2007 which is a breakthrough in targeted treatment for lung cancer. As the third kinase fusion gene in lung cancer, RET fusions own therapeutic significance, because they are targetable with several US Food and Drug Administration (FDA) approved multikinase inhibitors with anti-RET activity, including Vandetanib, Cabozantinib, Lenvatinib, Alectinib, Sunitinib, Regorafenib, and Sorafenib, with response rates ranging between 16% and 47% and median progression-free survivals (PFS) ranging from 2.3 to 7.3 months^7–10^.

The incidence and molecular characteristics of lung cancer in females are different from those in males. Similar to ALK and ROS proto-oncogene 1 (ROS1) fusions, RET fusion is likely to be specific to lung adenocarcinoma, and mainly detected in young female and/or never/light-smoking patients which is similar to ROS1 fusion^11–15^. Recently, a few researches have summarized the molecular characteristics and clinical features of patients with RET fusions. Shumei *et al*. investigated the molecular portfolio of 4,871 patients undergoing targeted next generation sequencing (NGS) and found RET fusions were detected in 1.8% of diverse cancers^16^. Michal *et al*. reported the response to therapy and clinical features in 14 patients with lung carcinoma harboring RET fusions^2,4,6,17^. However, no report on the genetic characteristic and clinical prognosis of RET fusions in female patients with lung cancer is available. Furthermore, there’s neither prospective clinical study nor successful case about immune checkpoint inhibitors (ICI) therapy in these patients.

Aiming to facilitate treatment targeting RET fusions, we analyzed the molecular portfolio of 1,652 patients with lung cancer who underwent targeted NGS, and revealed the genetic and clinical prognostic characteristics of female patients with lung cancer harboring RET fusion for the first time. In addition, we first reported the firstdelete this first patient with high expression of programmed death–ligand 1 (PD–L1) who responded favorably to nivolumab.

## Materials and Methods

### Patients

**Figure 1:**
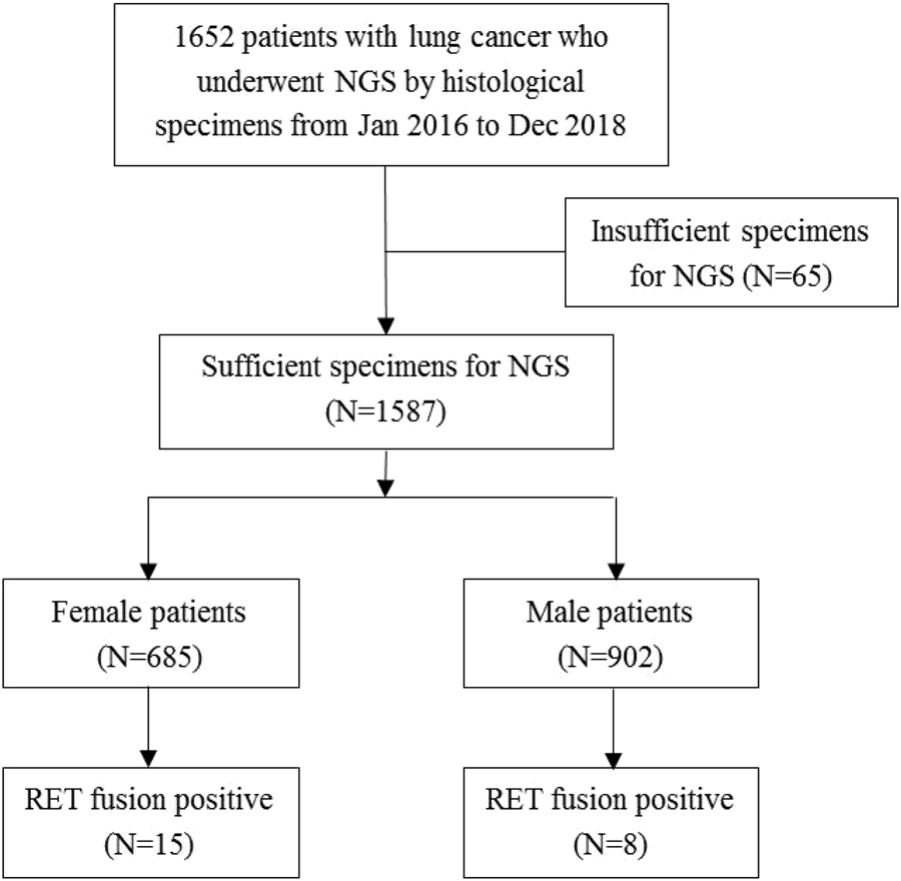
Patient screening flowchart.

1,652 patients with lung cancer who underwent targeted NGS by histological specimens from January 2016 to December 2018 were investigated (Fig. 1). Among them, 65 patients were excluded due to insufficient specimens for targeted NGS, hence 1,587 patients were included in this study finally. All these objects underwent surgical resection or tissue biopsy, and their tumors were diagnosed according to the *2015 World Health Organization (WHO) and the International Association for the Study of Lung Cancer (IASLC) Guidelines*. Clinical staging was based on the 8^th^ edition of the *TNM Classification for NSCLC*. The ethics of this study was approved by the Institutional Review Board of the West China Hospital, Sichuan University (approval No.: 2016-85instead of 2019-316). For the fulfillment of this retrospective study, written informed consents have been signed by all the patients, which enable us to access to case details and all accompanying images published.

### Tissue specimens and targeted NGS data analysis

We collected sequencing information from 1,587 patients whose tissue specimens had been reviewed to ensure tissue adequacy (surface area ≥25 mm^2^, volume ≥1 mm^3^, nucleated cellularity ≥80%, and tumor content ≥20%). DNAs were extracted with QIAamp DNA FFPE (Formalin-fixed and parrffin-embedded) Tissue Kit (Qiagen, California, USA) according to the manufacturer’s instructions. Targeted NGS library was prepared based on a previous publication^18^. Nucleotide fragments were selected and hybridized with capture probe baits. Genomic DNAs were profiled with a capture-based targeted sequencing panel, which is a commercially available panel by Burning Rock Biotech (Guangzhou, China). Selected exons and introns of 8/56 cancer-related genes was covered, spanning 170 kb of human genome. The median sequencing depth was 1855×. A bioanalyzer high sensitivity DNA assay was then used to evaluate their quality and size range. Available indexed samples were then sequenced with a Next-Seq 500 (Illumina, Inc., USA) by pair-end reads.

Targeted NGS data were mapped against the human genome (hg19) with BWA aligner 0.7.10 (http://bio-bwa.sourceforge.net/). Local alignment optimization, variant calling and annotation were then performed with GATK 3.2, MuTect, and VarScan successively. DNA rearrangement was analyzed by both Tophat2 and Factera 1.4.3^19^.

### Statistical analysis

Data were analyzed with Statistical Package for the Social Sciences (SPSS) software (Chicago, IL, USA, version 20.0). Between-group variations were compared by Pearson’s chi-squared test or Fisher’s exact test. Scatter dot plots and box & whisker plots were generated to indicate the medians and 95% confidence intervals (CI). A two-sided *P* ≤ 0.05 was considered statistically significant^19^.

## Results

### Analysis of RET fusions in lung cancer and patient characteristics

**Figure 2:**
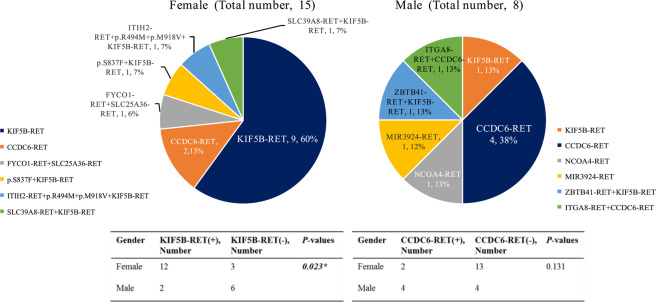
Gender ratios of RET gene fusions. Among the 1,587 patients, RET fusions were identified in 23 cases, 15 females and 8 males. The most common fusions were KIF5B–RET in females (*P* = *0.023*) and CCDC6–RET in males (*P* = *0.131*).

Among the 1,587 patients, RET fusions were identified in 23 cases [1.4% (23/1587)], 15 females [2.2% (15/685) and 8 males [0.9% (8/902)]. The most common fusions were KIF5B–RET in females [80% (12/15)] and CCDC6–RET in males [50% (4/8)]. Some rare RET fusions were also detected, such as SLC39A8–RET, ITIH2–RET, FYCO1–RET and SLC25A36–RET in females, and MIR392–RET, ZBTB41–RET and ITGA8–RET in males. Six patients were combined with tumor protein 53 (TP53) mutations [26.1% (6/23)], including one ROS1 mutation and one epidermal growth factor receptor (EGFR) mutation (Fig. 2).

Lung cancers involved in these 23 cases consisted of 19 adenocarcinomas (82.5%), two neuroendocrine carcinomas, one adenosquamous carcinomas, and one squamous cell carcinoma. The average age of these patients was 52.3 (31–77) years and only five men had smoking history. Immunohistochemical (IHC) results showed nine patients with positive expression of PD–L1 and one patient with ROS1 positive, and all ALK–V staining results were negative. Upper lobes were the most common location of lesions, and lobulated and spiculated were the most common imaging features. However, lymph node and pleural metastasis were often detected. Furthermore, serum tumor markers of cytokeratin 19 fragment (CYFRA21-1), carcino-embryonic antigen (CEA) and neuron specific enolase (NSE) in most patients increased obviously (Table 1).

**Table 1:** Summary of RET fusions in lung cancer and patient characteristics.

NO.	Panel	Gender	Age	Results of NGS		Pathology	PD–L1	Smoking	CT	TNM Stage	Therapeutic	PFS1 (Mo.)	DFS (Mo.)
				RET Fusion	Other Mutations								
1	56	F	54	SLC39A8-RET (S6:R12) Fusion KIF5B–RET (K22:R12) Fusion	KDR 16 Exon p.A757P Mutation ROS1 12 Exon p.R448H Mutation MTOR 39 Exon p.A1800V Mutation TP53 Mutation KDR 9 Exon p.K366E Mutation	ADC	50%	N	LUL	IVB	AC × 5 cycles Cabozantinib 80 mg qd × 57 days	4	/
2	56	F	49	KIF5B–RET (K19:R12) Fusion		ADC	0	N	RML	IB	Operation	/	>24
3	56	F	44	KIF5B–RET (K15:R12) Fusion	TP53 5 Exon p.C135F Mutation	ADC	90%	N	LUL	IVA	/	1	/
4	8	F	44	KIF5B–RET (KNA:R12) Fusion		Neuroendocrine carcinoma	0	N	RUL	IIA	Operation EP × 4 cycles	/	>30
5	56	F	53	KIF5B–RET (KIntron15:R11) Fusion	BRCA2 27 Exon Mutation TP53 5 Exon Mutation ATM 32 Exon p.R1633G Mutation	NSCLC	80%	N	RUL	IIIB	/	1	/
6	8	F	54	CCDC6–RET (C1:R12) Fusion		ADC	/	N	LUL	IIIA	Operation	/	>18
7	56	F	34	CCDC6–RET (C1:R12) Fusion		ADC	/	N	RUL	IA	Operation		>10
8	56	F	73	KIF5B–RET (K15:R11) Fusion	RET 14 Exon p.S837F Mutation	ADC	30%	N	RUL	IVA	Operation Traditional Chinese medicine	/	>17
9	8	F	54	KIF5B–RET (K19:R12) Fusion		ADC	0	N	LUL	IA	Operation	/	>19
10	56	F	54	ITIH2-RET (Iintergenic:R12) Fusion KIF5B–RET (K15:R12) Fusion	BRCA2 11 Exon p.T1154S Mutation RET 7 Exon p.R494M Mutation RET 16 Exon p.M918V Mutation	Adenosquamous carcinoma	0	N	LLL	IVB	Intralumen injection with platinu and bevacizumab	1.5	/
11	56	F	52	KIF5B–RET (K15:R11) Fusion		ADC	0	N	RUL	IA	Operation	/	>18
12	56	F	43	FYCO1-RET (F8:R12) Fusion SLC25A36-RET (S1:R12) Fusion		ADC	0	N	RUL	IA	Operation	/	>15
13	56	F	75	KIF5B–RET (K15:R12) Fusion	TP53 5 Exon Mutation ERBB4 24 Exon p.R983T Mutation MYC Amplification	ADC	/	N	LLL	IVA	Cabozantinib 60 mg qd × 30 days	7	/
14	8	F	39	KIF5B–RET (K15:R12) Fusion		ADC	0	N	RML	IVB	TC × 4 cycles	4	/
15	56	F	43	KIF5B–RET (K15:R12) Fusion		ADC	65%	N	RLL	IVA	AC × 4 cycles A × 2 cycles Nivolumab × 9 cycles	4.2	/
16	8	M	62	CCDC6–RET (C1:R12) Fusion	MET 14 Exon Mutation	Neuroendocrine carcinoma	10%	Y	RML	IVA	TC × 6 cycles Everolimus	14	/
17	8	M	46	NCOA4–RET (NIntron8:R12) Fusion		ADC	/	N	RUL	IVB	AP × 6 cycles γ knife × 14 times Intralumen injection with platinum	13	/
18	56	M	77	MIR3924-RET (Mintergenic:R3) Fusion	EGFR 21 Exon p.L861Q Mutation TP53 5 Exon Mutation EGFR 28 Exon Mutation TP53 5 Exon p.Y163C Mutation KRAS Amplification	SCC	0	N	RUL	IVA	Gefitinib Afatinib	5	/
19	56	M	35	ZBTB41-RET (Z9:R12) Fusion KIF5B–RET (K15:R12) Fusion	RB1 20 Exon Mutation TP53 5 Exon p.R181C Mutation	ADC	<5%	Y	RUL	IVA	Keytruda × 8 cycles AP + Bevacizumab × 4 cycles A + Bevacizumab+nivolumab	6	/
20	56	M	64	CCDC6–RET (C1:R12) Fusion	MTOR 1 Exon Mutation	ADC	5%	N	RML	IIIA	Operation TP × 4 cycles	/	>13
21	56	M	31	KIF5B–RET (K16:R12) Fusion		ADC	0	Y	LUL	IA	Operation	/	>12
22	56	M	61	CCDC6–RET (C1:R12) Fusion		ADC	5%	Y	RLL	IIA	Operation	/	>11
23	56	M	62	ITGA8-RET (I30:R12) Fusion CCDC6–RET (C1:R12) Fusion		ADC	0	Y	LLL	IVB	AC × 2 cycles	6	/

F, female; M, male; ADC, adenocarcinoma; SCC, sequamous carcinoma; Y, yes; N, never; RUL, right upper lobe; RML, right middle lobe; RLL, right lower lobe; LUL, left upper lobe; LLL, left lower lobe; A, pemetrexed; C, carboplatin; E, etoposide; P, cis-platinum; T, paclitaxel; Mo., month. SLC39A8-RET (S6:R12) Fusion, intron 6 of SLC39A8 was ligated to intron 11 of RET.

### Expression of PD–L1 in female patients

**Figure 3:**
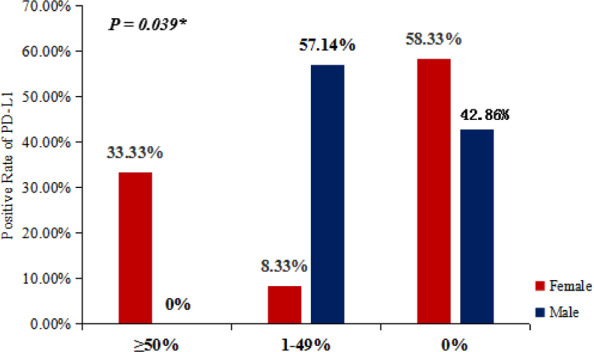
Positive rates of PD–L1 in females and males. In females, the highly positive (≥50%), moderate positive (1–49%), and negative (0%) rates of PD–L1 staining were 33.3%, 8.3% and 58.3%, respectively, whereas those in males were 0%, 57.1% and 42.9%.

Nine (five women and four men) out of 23 patients had tumors with positive expression of PD–L1 (Antibody, 22C3, Dako Link 48, Agilent). Interestingly, the highly positive, moderate positive and negative rates of PD–L1 staining in females were 33.3%, 8.3% and 58.3%, respectively, whereas those in males were 0%, 57.1% and 42.9%. Women are more likely to have high expression of PD–L1 than men (Fig. 3*, P* = 0.039). Additionally, we found that KIF5B–RET fusion was detected in all female patients with high expression of PD–L1; while CCDC6–RET fusion was detected in all male patients with positive expression of PD–L1.

### Prognosis of lung cancer patients with RET fusions

**Figure 4:**
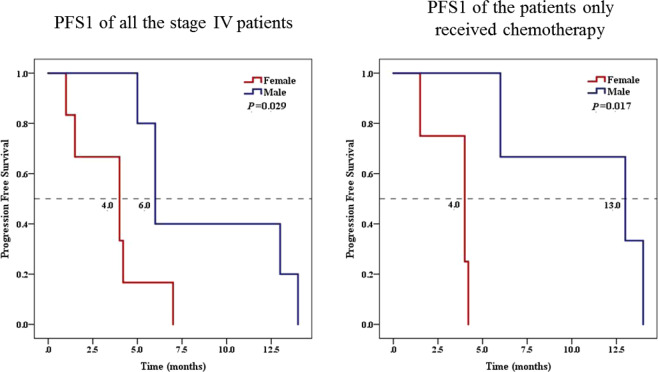
Progression free survivals in stage IV patients and patients only receiving chemotherapy. In stage IV patients, the median PFSs were 4.0 months in females and 6.0 months in males (*P* = *0.029*). The median PFSs of patients only receiving chemotherapy were 4.0 months in females and 13.0 months in males (*P* = *0.017*).

We analyzed the PFSs of all stage IV lung cancer patients and those only receiving chemotherapy, respectively. In the stage IV patients, women had shorter PFS than men, shown by the medians of 4.0 months and 6.0 months respectively (*P* = 0.029). Moreover, this feature was more apparent among patients only receiving chemotherapy, the medians were 4.0 months in females and 13.0 months in males (*P* = 0.017) (Fig. 4).

### Brief case report

**Figure 5:**
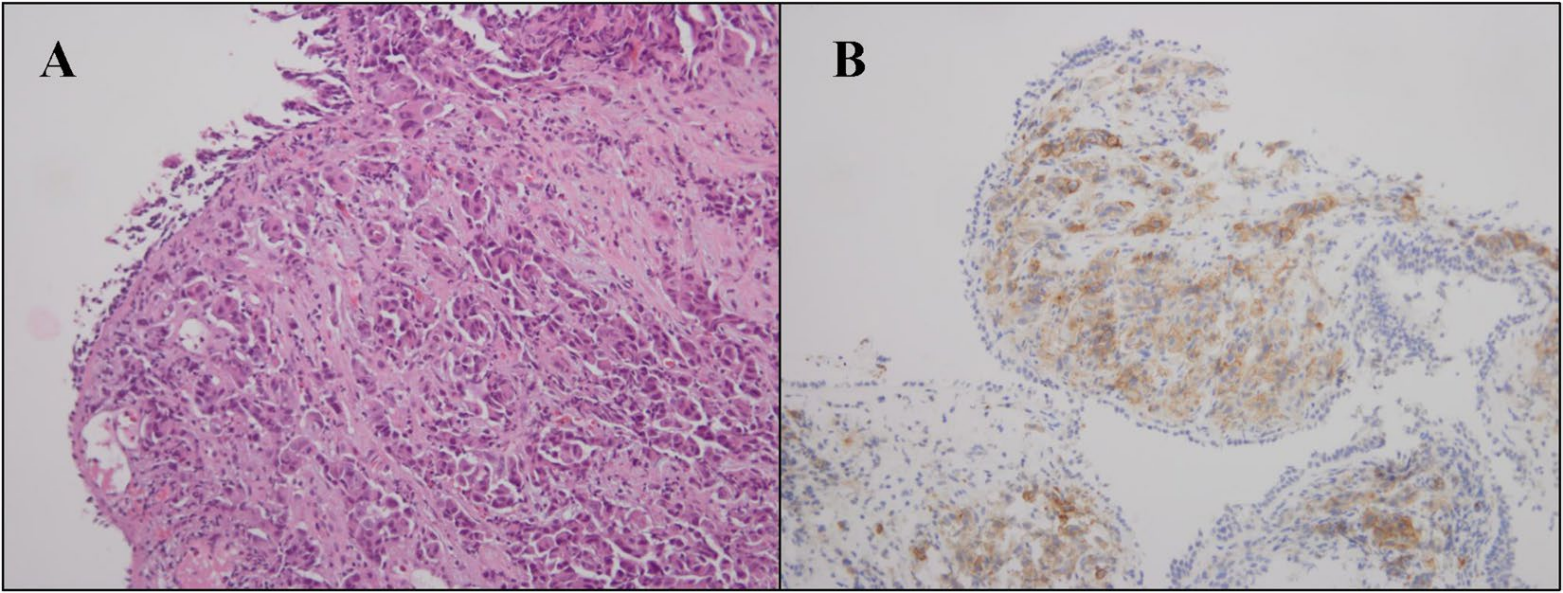
IHC staining of HE and PD-L1. HE staining, B. IHC staining demonstrated PD–L1 (+, positive rate of about 65%), magnification ×200.

A 43-year-old female nonsmoker was admitted to our Lung Cancer Center due to cough and dyspnea. CT (Computed tomography) of the chest found a mass in the right upper lobe with right hilum and mediastinal lymph node enlargement, as well as a lesion in the left frontal lobe considered as metastasis. Adenocarcinoma was identified by fiberoptic bronchoscopy. Immunohistochemical (IHC) staining demonstrated ALK–V (−), ROS1 (−) and PD–L1 (+, positive rate of about 65%) (Fig. 5). As a result, she was diagnosed with stage IV poorly differentiated adenocarcinoma of the lung. Targeted NGS revealed a KIF5B–RET fusion.

**Figure 6:**
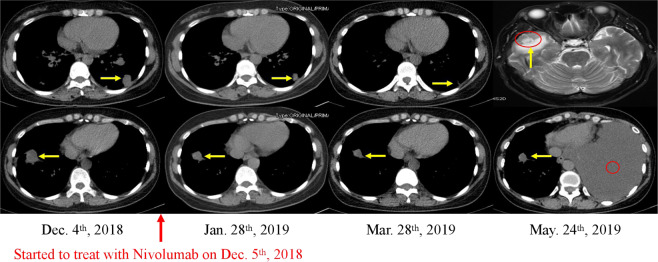
Imaging changes in a patient treated with nivolumab. A 43-year-old female nonsmoker was diagnosed with stage IV poorly differentiated adenocarcinoma of the lung. Targeted NGS revealed a KIF5B–RET fusion. Her disease progressed after 4.2 months of platinum-based chemotherapy, and then became partially respond to second-line treatment with nivolumab. However, new lesions of her disease were detected after 5.5 month.

Her disease progressed after 6-cycle first-line treatment of pemetrexed plus carboplatin. A new lesion appeared in the left lower lobe and the result of efficacy evaluation was PD (progressive disease, RECIST 1.1). Considering the high expression of PD–L1, we decided to discontinue the chemotherapy and began to use nivolumab (Opdivo; Bristol-Myers Squibb, New York, NY). Subsequently, she was started on nivolumab 3 mg/kg every 14 days. To our surprise, the sizes of the masses on both sides have reduced significantly, especially the left one, after 9 cycles. However, new lesions (Intracranial metastasis and left pleural effusion)were detected after 5.5 months (Fig. 6). Patient experienced fatigue and occasional cough during the treatment.

## Discussion

To our knowledge, this is the first study revealing the genetic characteristics of RET fusions in female patients with lung cancer. RET fusions were present in 2.2% of female lung cancers that was higher than that in males (0.9%). The most common fusion in females was KIF5B–RET (80%), whereas that in males was CCDC6–RET (50%). All of the three male patients harboring CCDC6–RET fusion had smoking history, thus we suspected CCDC6–RET fusion was associated with smoking. Shumei *et al*. analyzed RET aberrations in 4,871 patients with diverse cancers by NGS, and found RET fusions were present in 3.3% of lung cancer^16^. Gou *et al*. reported that RET mutations were identified in 1.6% of Chinese NSCLC^3^. Other studies showed RET rearrangements occurred in approximately 2% of lung adenocarcinoma and in 1% of all lung cancers^20^. In concordance with previous reports, the rate of RET fusions in all lung cancers was 1.4% in our results.

However, all the previous researches have not shown the genetic characteristics of female patients with lung cancer harboring RET fusions. Our study revealed the gender ratio of RET gene fusions for the first time. To date, many RET fusions have been reported in NSCLC, including KIF5B–RET, CCDC6–RET, NCOA4–RET, TRIM33–RET, RUFY2–RET, CUX–RET, KIAA1468–RET, CLIP1–RET, ERC1–RET, MYO5C–RET, EPHA5–RET, PICALM–RET, FRMD4A–RET, KIF13A–RET and WAC–RET^17^. We also found several rare RET fusions including SLC39A8–RET, ITIH2–RET, FYCO1–RET and SLC25A36–RET in females and MIR3924–RET, ZBTB41–RET and ITGA8–RET in males, which may have not been reported before.

In our study, an interesting phenomenon was discovered for the first time, women were more likely to have high expression of PD–L1 (≥50%) than men (*P* = 0.039); furthermore, all female patients with high expression of PD–L1 harbored KIF5B–RET fusion, while all male patients with positive expression of PD–L1 harbored CCDC6–RET fusion. Some researches revealed the presence of squamous cell carcinoma (SCC), wild-type EGFR or KRAS gene mutations was associated with high expression of PD–L1, providing potentially benefits for the administration of PD-1/PD–L1 blockade in lung cancer^21^. Miura Y *et al*. identified smoking as a predictive marker of response to immune checkpoint inhibitors (ICIs), showed that nonsmokers with EGFR mutated or ALK rearranged tumor did not respond well to ICIs, and suggested patients should not receive ICIs as first-line treatment even though high PD–L1 expression was detected in their tumor cells^22^. The use of ICIs remains controversial in second line. The KEYNOTE-010 and CheckMate 057 trials, which tested the benefits of pembrolizumab and nivolumab were over docetaxel chemotherapy in second line, didn’t show any differences between EGFR-mutant and ALK-fusion patients^23,24^. However, recent results from the IMpower150 trial may change treatment option for patients with tyrosine kinase inhibitor (TKI) relapse. This trial included EGFR and ALK positive patients whose diseases had progressed after TKI and indeed showed a PFS benefit in the atezolizumab + chemotherapy + bevacizumab group compared with the chemotherapy + bevacizumab group, with a mean PFS of 9.7 versus 6.1 months^25,26^. The clinical value of TKIs-ICIs combination therapy remains controversial, for some results showed the severe side effects. Lung cancers frequently exhibit high tumor mutational load (TML) related to tobacco exposure. Rizvi *et al*. using WES demonstrated on a small series of patients that TML was predictive for ICIs treatment response. So, TML has become a predictive marker for immunotherapy^27,28^. How about immunotherapy for lung cancer patients with RET fusion? For now, no report on prospective clinical study or successful case is available. Michal Sarfaty *et al*. reported two patients’ tumors showed PD–L1 positive (≥50%) staining but did not respond to pembrolizumab. Both of the two cases were female nonsmokers with KIF5B–RET fusion, and the TMLs were very low in one patient and intermediate in the other^4^. Here, we also, for the first time, reported a 43-year-old female nonsmoker diagnosed with stage IV poorly differentiated adenocarcinoma of lung harboring KIF5B–RET fusion with highly positive PD–L1 staining (65%). After the second-line treatment with nivolumab, a partial response was achieved along with a PFS of more than 5 months. It prompts that immunotherapy may be a treatment option for this type of patients, which still needs more clinical practices to verify. In fact, there have been also some clinical trials of RET inhibitors such as selpercatinib, cabozantinib, vandetanib and alectinib^29^. Drilon *et al*. conducted a prospective phase 2 trial in patients with RET-rearranged lung cancers, and found that the overall response rate (ORR) was 28% [95% CI 12–49%], but 73% of the patients required dose reduction due to drug-related adverse events^10^. Nokihara *et al*. evaluated cabozantinib in Japanese patients with advanced solid tumors, and recommended the maximum tolerated dose of cabozantinib was 60 mg daily, and the recommended phase 2 dose of cabozantinib was determined to be 60 mg daily. Moreover, the conditions of two patients with RET fusions remained stable and showed best response to cabozantinib^30^. In the phase I/II LIBRETTO-001 trial, the experimental RET inhibitor selpercatinib (LOXO-292; Eli Lilly) elicited high response rates lasting more than a year and a half in patients with RET-altered NSCLC who had already received multiple treatments. The median PFS reached 18.4 months that is indeed longer than that of our case^31^. No indication for any RET inhibitor is approved and no RET inhibitor is available for sale in China, though selpercatinib has become the first RET inhibitor on the path to FDA approval. Actually, we are conducting a phase I/II clinical trial of BLU-227 in China. We believe that RET inhibitors have definite curative effect on patients with lung cancers carrying RET fusions; however, due to their many side effects, it is a research trend to develop inhibitors with more specific targets. Because no RET inhibitor has been indicated for the treatment of lung cancer in China yet, and the patient in our case had obtained PR to immunotherapy with a PFS of more than 5 months, so immunotherapy can still be a treatment option for patients with RET fusion lung cancer at present.

In addition, Wang *et al*. showed lung adenocarcinomas patients with RET fusion gene had more poorly differentiated tumors and showed a tendency to be younger and nonsmokers and to have a smaller tumor (≤3 cm) with N2 disease^15^. Michal Sarfaty *et al*. showed clinical features of 14 lung cancer patients with RET fusions, whose median overall survival was 22.8 months, in which, that of the patients whose tumor harbor KIF5B–RET subtype was 13.3 months, compared with 22.8 months for CCDC6–RET subtype^4^. It is shown in our results, PFS is significant shorter in females than that in males no matter in the stage IV group or in the group only receiving chemotherapy (all *P* ≤ 0.05). This is probably because 80% of the female patients carries KIF5B–RET fusion in our study, which may point out a more aggressive biological behavior of KIF5B–RET subtype.

In summary, based on our data, RET fusions occur in 2.2% of female patients with lung cancers. KIF5B–RET is the most common subtype in females, often combined with high expression of PD–L1; and female rather than male patients harboring KIF5B–RET fusion are prone to poor prognosis. Immunotherapy may be a treatment option, but it needs more clinical practices.
